# Does Village Chicken-Keeping Contribute to Young Children’s Diets and Growth? A Longitudinal Observational Study in Rural Tanzania

**DOI:** 10.3390/nu10111799

**Published:** 2018-11-19

**Authors:** Julia de Bruyn, Peter C. Thomson, Ian Darnton-Hill, Brigitte Bagnol, Wende Maulaga, Robyn G. Alders

**Affiliations:** 1 Natural Resources Institute, University of Greenwich, Kent ME4 4TB, UK; 2 School of Life and Environmental Sciences, University of Sydney, Sydney NSW 2006, Australia; peter.thomson@sydney.edu.au (P.C.T.); robyna@kyeemafoundation.org (R.G.A.); 3 Charles Perkins Centre, University of Sydney, Sydney NSW 2006, Australia; ian.darnton-hill@sydney.edu.au (I.D.-H.); bagnolbrigitte@gmail.com (B.B.); 4 The Boden Institute of Obesity, Nutrition, Exercise & Eating Disorders, University of Sydney, Sydney NSW 2006, Australia; 5 Department of Anthropology, University of Witwatersrand, Johannesburg 2000, South Africa; 6 International Rural Poultry Centre, Kyeema Foundation, Brisbane QLD 4000, Australia; 7 Tanzania Veterinary Laboratory Agency, Dar es Salaam 11000, Tanzania; wendesamanga@gmail.com

**Keywords:** undernutrition, food security, nutrition security, village chickens, livestock, animal-source food, Tanzania, sub-Saharan Africa, resource-poor settings

## Abstract

There is substantial current interest in linkages between livestock-keeping and human nutrition in resource-poor settings. These may include benefits of improved diet quality, through animal-source food consumption and nutritious food purchases using livestock-derived income, and hazards of infectious disease or environmental enteric dysfunction associated with exposure to livestock feces. Particular concerns center on free-roaming chickens, given their proximity to children in rural settings, but findings to date have been inconclusive. This longitudinal study of 503 households with a child under 24 months at enrolment was conducted in villages of Manyoni District, Tanzania between May 2014, and May 2016. Questionnaires encompassed demographic characteristics, assets, livestock ownership, chicken housing practices, maternal education, water and sanitation, and dietary diversity. Twice-monthly household visits provided information on chicken numbers, breastfeeding and child diarrhea, and anthropometry was collected six-monthly. Multivariable mixed model analyses evaluated associations between demographic, socioeconomic and livestock-associated variables and (a) maternal and child diets, (b) children’s height-for-age and (c) children’s diarrhea frequency. Alongside modest contributions of chicken-keeping to some improved dietary outcomes, this study importantly (and of substantial practical significance if confirmed) found no indication of a heightened risk of stunting or greater frequency of diarrhea being associated with chicken-keeping or the practice of keeping chickens within human dwellings overnight.

## 1. Introduction

To achieve lasting impact in enhancing food and nutrition security in resource-poor settings, there is a need for strategies which align with local priorities and address context-specific constraints. Mixed crop-livestock farming is widely practiced throughout sub-Saharan Africa [1] and has been shown to strengthen the resilience of smallholder farmers to climate change [2,3]. A growing body of literature explores linkages between livestock ownership and human nutrition. Livestock-keeping is posited to offer the benefits of improved dietary quality, through access to nutrient-rich animal-source foods (ASF) [4,5] and nutritious food purchases enabled by livestock-derived income [6], but with the possible hazards associated with exposure to livestock feces in rural settings [7].

Potential risks of contact with livestock feces relate both to pathogenic bacteria, responsible for infectious diarrhea [8], and to non-pathogenic bacteria, as a cause of environmental enteric dysfunction (EED) [9]. In EED, chronic damage to the gastrointestinal tract may reduce nutrient absorption and cause low-level immune stimulation, restricting children’s growth and development [10,11]; however, evidence of the causal pathways between EED and child stunting is limited, and the condition is likely to be more complex than previously conceived [12]. A systematic review examining relationships between livestock ownership and child health identified a predominant focus on diarrhea as the primary health outcome [8]. Impaired height-for-age (stunting) is advocated as a more objective measure which reflects the cumulative effect of multiple influences, such as dietary quality and recurrent diarrhea, on children’s growth over an extended period of time [13]. 

Village chickens are an accessible and versatile form of livestock, kept in small flocks by many vulnerable households in low- and middle-income countries (LMICs). Indigenous-breed birds roam freely during the day, scavenging for food, and are often housed within human dwellings overnight to reduce risks of predation or theft [14,15]. Their contribution to income and diets is made significant by the inherently low requirements for capital, labor and other inputs [15,16,17]. These benefits may be further enhanced by the central role of women in raising village chickens [18,19,20], with income and resources managed by women shown to have disproportionately strong effects on health and nutrition [21,22]. While previously promoted as an opportunity to sustainably enhance food and nutrition security in LMICs, free-ranging chickens have faced recent scrutiny as a source of fecal contamination in rural areas.

Recent efforts to determine the net impact of chicken-keeping on children’s growth and health have largely reported on the analysis of existing datasets [23,24,25,26]. These multi-purpose surveys have the advantage of large sample sizes, but offer a limited opportunity to understand specific management practices which may carry benefits and risks to human health and nutrition. In Ethiopia, the practice of keeping chickens indoors overnight was reported to counteract any positive effects of poultry ownership on children’s height-for-age Z-scores (HAZ) [27]. A multi-country study found animal feces in the homestead environment to be negatively associated with children’s HAZ in Bangladesh and Ethiopia, but not in Vietnam [24].

Appropriate source data is vital to draw meaningful conclusions amidst the complexity of different livestock production systems, sociocultural influences, and seasonal variation. Some studies have employed the Tropical Livestock Unit as a quantitative measure to evaluate livestock ownership as a predictor of nutrition outcomes [24,28]. This unit represents smallholder livestock holdings—of mixed species, breeds and ages—based on the approximate metabolic weight of each. This unit was developed to quantify production levels and relate livestock numbers to human populations or land resources [29,30]. There are clear limitations in its use in situations where the “amount” of livestock may not equate, for example, to their sociocultural value or to the incidence of stunting, wasting or illness in children.

The concept of the “livestock ladder” has been used to describe livestock ownership as an opportunity for rural households to accumulate assets and rise from poverty [31,32,33]. Beyond their economic value, however, there are notable distinctions in the roles of different livestock in resource-poor settings. Village chickens, considered the lowest step on the livestock ladder, offer a readily-accessible source of income, an opportunity to engage in social customs, and access to nutritious food items [20,34,35]. By contrast, larger livestock such as cattle are associated with wealth and social status, and provide longer-term savings, insurance, and draught animal power, but are infrequently used to meet immediate household needs [32,36,37]. 

This paper presents analytical approaches and results from an observational study in eight rural communities in Tanzania. Alongside other forms of livestock ownership, it includes a specific focus on village chicken-keeping to evaluate longitudinal associations with (a) diets of young children and their mothers, (b) HAZ and the probability of stunting in children, and (c) the occurrence of diarrhea in children. The study is nested within a larger investigation assessing the impact of community-based vaccination programs against Newcastle disease in village chickens and interventions to improve crop diversity, management, and storage on maternal and child health outcomes [38]. Analyses have used a range of livestock-associated predictor variables, and both short-term (i.e., diets and diarrhea) and longer-term (i.e., HAZ) outcome variables, to optimize the understanding of how domestic animal ownership has influenced children’s nutrition and health over a two-year period in this setting. 

## 2. Materials and Methods 

### 2.1. Study Area and Population

This longitudinal study of 503 children was conducted in eight rural villages in Manyoni District, Singida Region in the semi-arid Central Zone of Tanzania. Unimodal rainfall is expected between November and April, with mean annual rainfall of 624 mm (SD = 179 mm) and a mean of 49 rain days per year (SD = 15) at a district level [39]. Project sites were selected in consultation with government partners at national, regional and district levels, guided by the prevalence of childhood stunting and the absence of existing nutritional interventions. 

A ward census was conducted by the project in April 2014 in Sanza Ward and October 2014 in Majiri Ward, with local enumerators visiting all households in each community to record the age and gender of all members, based on information provided by a household representative. Two-stage sampling was used to enroll a total of 240 households in Sanza Ward and 280 in Majiri Ward, by first enrolling all households with a child under 12 months of age (mo), and then using random selection to enroll additional households with a child aged 12–24 mo. The terms “enrolled child” and “enrolled household” are used to describe participants in this study, with a single child followed within each household (the youngest child, where more than one child under 24 mo was present).

Baseline data collection was completed for 229 households from Sanza Ward in May 2014, and 274 households from Majiri Ward in November 2014, as part of the staged implementation within the larger project design [38]. A small number of enrolled children (*n* = 6) were identified to be above the intended maximum age of 24 months at this time (range of 24.2–28.1 mo), but were retained within the study sample. 

### 2.2. Ethical Approval

Study design, protocols and research tools were approved by the Tanzanian National Institute for Medical Research ethics committee (NIMR/HQ/R.8a/Vol.IX/1690), and by the University of Sydney’s Human Research Ethics Committee (2014/209) and Animal Ethics Committee (2013/6065). Participants’ informed consent was given via a signature or thumb print at the time of data collection, for each questionnaire and set of anthropometric measurements. A participant information statement was provided to all participating households at the commencement of the study. To accommodate linguistic diversity and varying levels of literacy, all documents were read aloud to study participants by trained enumerators, using local languages where appropriate.

### 2.3. Data Collection

#### 2.3.1. Questionnaires

Male and female enumerators were recruited from the community in consultation with local leaders and trained to administer two semi-structured questionnaires. One questionnaire, directed to mothers at six-monthly intervals, encompassed maternal education, household water sources and toilet facilities, and maternal and child diets (24-h dietary recall, using the “open recall” method [40]). A second questionnaire, applied annually to an intended equal number of male and female household members (actual sample comprised 60.5% female respondents of 1354 completed questionnaires) encompassed demographic data, household assets, livestock ownership and chicken-keeping practices, including the location of overnight housing for chickens. Printed survey questions and training sessions were in Swahili, but enumerators were encouraged to make use of the languages of the two predominant language groups (*Kigogo* and *Kisukuma*) where appropriate.

#### 2.3.2. Household Visits

Male and female representatives from each village were employed as “Community Assistants” to collect ongoing data. Households were visited twice-monthly to record the number of chickens owned (categorized by age, i.e., under or over two months), the breastfeeding status of the enrolled child (exclusively breastfed, receiving both breast milk and complementary foods, or non-breastfed), and the occurrence of diarrhea in the enrolled child during the previous two weeks. Diarrhea was defined as the passage of three or more loose or liquid stools per day, or more frequent passage than is normal for the individual child [41].

#### 2.3.3. Anthropometry

Child length or height measurements were recorded to the nearest 1 mm by trained personnel using UNICEF portable baby/child length-height measuring boards. Recumbent length was measured for children up to 24 mo, and standing height for children over 24 mo. Where this protocol was not followed, to minimize distress and maximize measurement accuracy (6.0% measurements), a standard adjustment was applied [42]. Birthdates were verified against health clinic records where available (80.7%). Measurements were taken at six-monthly intervals from May 2014 in Sanza and November 2014 in Majiri until May 2016 in both wards.

#### 2.3.4. Rainfall Data

Daily rainfall data were recorded by trained community representatives from a rain gauge with 1 mm graduations, located at a village office in each ward. 

### 2.4. Data Analysis

#### 2.4.1. Defining Variables

Descriptions and data sources for predictor and outcome variables are provided in Appendix A (Table A1). Emergency Nutrition Assessment for SMART software was used to calculate child HAZ, based on World Health Organization (WHO) child growth standards [42]. Z-scores below −6 or above +6 were identified as extreme or potentially incorrect values, and were excluded from analyses [43]. Z-scores of less than −2 for height-for-age were classified as stunting. A “diarrhea score” was calculated for each six-month period preceding anthropometry, from a ratio of the number of positive records of diarrhea to the total number of records per child. This allowed for variability in the number of data points per child, due to absence during data collection visits. 

For mothers, dietary diversity (DD) scores were based on the Minimum Dietary Diversity for Women of Reproductive Age (MDD-W) indicator [40] and for children, using the Infant and Young Child Minimum Dietary Diversity (IYCMDD) indicator [44]. To achieve consistency in the longitudinal evaluation of children’s diets, the IYCMDD indicator was used beyond its intended age range of 6–23 months, as has been done elsewhere [45]. Results for breastfed and non-breastfed children are reported separately. Consumption of ASF, overall and in the categories of eggs, chicken meat, other meat and fish, and milk, was also evaluated. 

A household was defined as a group of people living together and sharing food from the same pot, with members having lived in the household at least three days of each week for the previous six months [46]. This definition seeks to encompass individuals who share common resources and make common budget and expenditure decisions. Given the close linkages between livestock and wealth in agropastoralist communities, multiple approaches were employed to assess the influence of livestock-keeping while controlling for variation in wealth. 

Socioeconomic status was represented using several variants of an index developed for use in sub-Saharan Africa, which assigns a weight to livestock and non-livestock assets according to their relative value [47]. Information on the development and validation of this index is not readily-available, but it is recommended for all projects receiving funding from the Bill and Melinda Gates Foundation. Guided by Tanzanian research partners and time in the study sites, the authors judged this tool to provide an adequate estimation of wealth in this setting. The index was modified to accommodate available data, and a weighting was applied to 11 household assets (radio, television, refrigerator, mobile phone, mosquito net, table, sewing machine, bicycle, motorcycle, car, and ox-cart) and six forms of livestock (cattle, sheep, goats, donkeys, pigs and poultry). Three variants of this index were used, as outlined in Table 1.

Information on the first language spoken by both of the enrolled child’s parents was collated with the gender of the household head to determine the dominant language group of each household, as a proxy for a range of cultural and agricultural practices [48,49]. Toilet facilities and sources of drinking water were classified as improved or unimproved [50].

To address the complex linkages between livestock, health and nutrition, multiple variables based on livestock ownership were assembled (Table A1). For each major category of animals, variables were constructed to reflect ownership:

(a) as a bivariate categorical variable (i.e., yes/no);
(b) relative to the median number of animals in this population (i.e., </≥ median number); and,
(c) in terms of the number of animals owned.

A “livestock ladder” variable was also calculated, which assigned households to four categories based on the animal species owned:

(a) no livestock;
(b) chickens only;
(c) small ruminants, with or without chickens; and,
(d) cattle, with or without other livestock.

**Figure 1:**
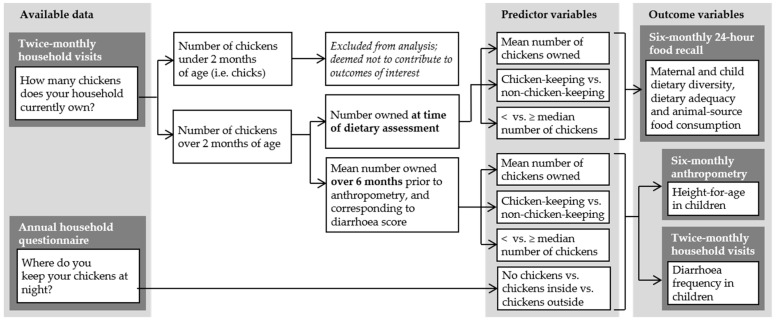
Construction of predictor variables to test associations between chicken-keeping and maternal and child diets, child anthropometry and diarrhea frequency (alongside other livestock and non-livestock variables). Small chicks have been excluded from chicken flock size, and consideration given to the time period over which ownership is measured.

Variables were constructed to reflect ownership for the six month period preceding each round of anthropometry. Information on ruminant ownership was drawn from the annual household questionnaire, but variables relating to chicken ownership were based on twice-monthly household visits. This reflects the substantial fluctuations in chicken ownership and flock size in village settings due to short reproductive cycles, sales to meet household needs, and losses due to predation or seasonal disease outbreaks. To evaluate contributions to diets, income and household environments, chicks were excluded from measures of chicken flock size. A summary of the construction of chicken-associated variables is shown in Figure 1. 

To test associations with diets, chicken ownership during the month of dietary assessment was used. For the longer-term outcomes of children’s HAZ and diarrhea, a broader assessment of chicken ownership was considered more relevant than a single point-in-time count of chicken numbers, therefore, the mean number of chickens owned during the six-month period preceding each measurement was calculated. Households were classified as “chicken-keeping” if the mean number of chickens owned was one or greater for a six-month period, and “non-chicken-keeping” if less than one. 

#### 2.4.2. Descriptive Statistics

Descriptive analyses were used to characterize the study population, explore variation between the two wards, and evaluate intended predictor and outcome variables. Percentages were determined for categorical variables, and means and standard deviations or medians and interquartile ranges calculated, for normally and non-normally distributed continuous variables, respectively. Inter-group comparisons for the two wards were performed using *t-*tests and chi-square tests for continuous and bivariate categorical variables, respectively. 

#### 2.4.3. Univariable and Multivariable Models

All analyses were conducted in the form of linear mixed models or generalized linear mixed models, using Genstat Release 18 software. Consideration was given to the potential for spatial clustering by including ward, village, and sub-village locations as random effects, along with household identifiers to account for repeat-measures data. Three broad components of the study evaluated:

(a) maternal and child dietary diversity, dietary adequacy and the consumption of ASF;
(b) child HAZ and probability of the stunting; and,
(c) children’s “diarrhea score”.

In each case, predictor variables of interest were collated for six-month periods corresponding to outcome variables. For livestock numbers and asset scores, log-transformations were used to minimize the excessive influence of very large numbers. Univariable models were first used to test unconditional associations between predictor and outcome variables. Multivariable models were constructed using variables of suggestive significance (*p* < 0.1) based on univariable models, and stepwise backward elimination was used to manually remove variables with *p*-values greater than 0.1 to reach the final models. Both significant (*p* < 0.05) and suggestive (0.05 ≤ *p* < 0.1) associations have been reported. 

## 3. Results

### 3.1. Characterizing the Population 

Of a total of 513 children randomly selected to participate in the study, adequate baseline data were available for inclusion of 503 children in this analysis. An attrition rate of 16.9% was seen at the time of final data collection in May 2016, due to relocation outside the study area (*n* = 39), withdrawal from the study (*n* = 5), and child deaths (*n* = 6). Table 2 presents an overview of individual and household characteristics at the time of baseline data collection, by ward and in the overall sample. At enrolment, the mean age of children was 8.6 mo (range of 0.6–28.1 mo). Almost one-third of mothers (32.5%) reported having had no formal education, and only a small number (3.4%) indicated a level of education beyond primary school.

Of approximately 120 language groups within Tanzania [51], 22 were represented within the study population. *Kigogo* was the primary language for 75.7% of households and *Kisukuma* the primary language for 9.1%. For a large majority of enrolled children, both parents shared their first language (94.1%). Households included a mean number of 5.5 members (range of 2–21), and 22.7% reported a female head of household. Less than 5% of households reported accessing an improved water source at the time of baseline data collection, less than 2% used improved toilet facilities, and almost three-quarters (72.7%) shared toilet facilities with one or more other households.

### 3.2. Livestock and Household Wealth 

The index of livestock and non-livestock assets (HDAI) varied widely across the study sample, with a markedly positively-skewed distribution. The median HDAI in Majiri Ward was more than twice that in Sanza Ward (26 vs. 12), while wealth assessments based on non-livestock assets varied less prominently. Ruminants were more commonly kept in Majiri than in Sanza (36.2% vs. 26.7%, *p* = 0.024 for cattle; 47.8% vs. 27.1%, *p* < 0.001 for sheep and goats), and cattle numbers significantly greater (median herd size of 10 in Majiri vs. 4 in Sanza, *p* = 0.012). Baseline data suggested chicken-keeping to be more common in Sanza than in Majiri, however the differing months of data collection and seasonal variation in chicken numbers prevents conclusions being drawn using these data. 

Based on data collated over the entire study period, 70.8% of households owned some form of livestock, with chickens kept by 55.0%, sheep and goats by 38.4% and cattle by 31.5%. Almost two-thirds of chicken-keeping households (64.2%) reported chickens to be kept inside their home overnight, rather than in a chicken house or left to roost in trees. Categorizing livestock ownership according to the “livestock ladder”, around one-quarter of participating households (25.3%) kept only chickens; 14.0% kept sheep or goats, with or without chickens but without cattle; and 31.5% kept cattle, with or without other livestock.

**Figure 2:**
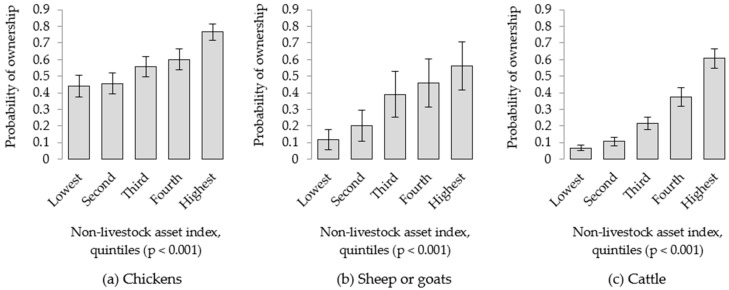
Probability of owning livestock, according to quintiles of the non-livestock asset index (NLAI). For all categories of animals—(**a**) chickens, (**b**) sheep and goats, and (**c**) cattle—the NLAI was positively associated with the probability of ownership (*p* < 0.001). Standard errors are shown.

Accounting for geographic clustering and individual household effects, the non-livestock index (NLAI) was positively associated (*p* < 0.001) with the probability of owning each category of animals—however the extent of this association varied between species (Figure 2). Households in the lowest quintile were identified to have a 0.44 probability of owning chickens, compared with a 0.12 probability of owning sheep or goats and a 0.07 probability of owning cattle. The comparative increase in the likelihood of animal ownership with increasing non-livestock wealth also varied between livestock categories. A household in the highest quintile had 21.2 times greater odds of owning cattle, 9.6 times greater odds of owning sheep or goats, and 4.1 times greater odds of owning chickens, compared to a household in the lowest quintile. 

**Figure 3:**
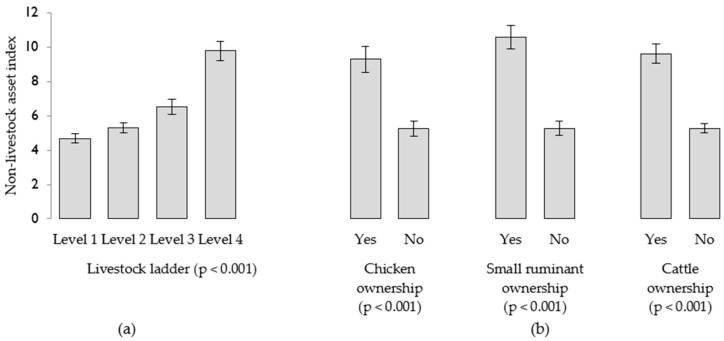
Model-based mean non-livestock asset index scores according to: (**a**) levels on the livestock ladder, and (**b**) ownership of chickens, sheep or goats, and cattle. Standard errors are shown.

Positive associations were also found between the livestock ladder and the NLAI (*p* < 0.001). Model-based means show markedly greater non-livestock wealth amongst households in the highest tier of the livestock ladder (i.e., those owning cattle), compared to other groups (Figure 3a). Ownership of chickens, small ruminants and cattle was associated with substantially higher mean non-livestock wealth scores, compared to not owning each form of livestock (Figure 3b).

### 3.3. Maternal and Child Diets 

#### 3.3.1. Dietary Diversity and Animal-Source Food Consumption

**Figure 4:**
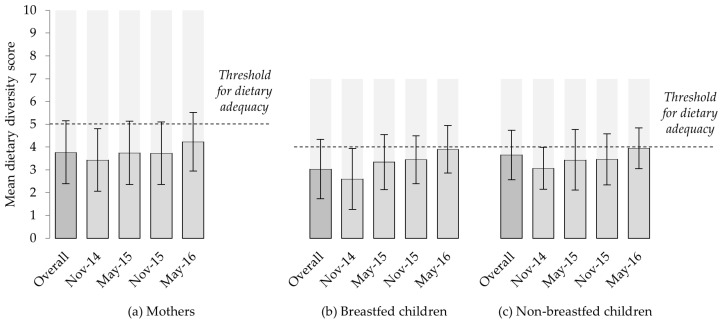
Mean dietary diversity scores of (**a**) mothers (using MDD-W indicator); and (**b**) breastfed and (**c**) non-breastfed children (both using IYCMDD indicator), overall and for each six-monthly data collection. Light grey shading indicates the number of food groups. Standard errors are shown.

The mean DD score across all data collection periods was 3.8 (SD 1.4) for mothers, 3.0 (SD 1.3) for breastfed children and 3.7 (SD 1.1) for non-breastfed children (Figure 4). Despite similarity in the number of food groups eaten, the percentage of non-breastfed children meeting cut-offs for an “adequately diverse” diet (55.6%, based on ≥ 4 of 7 food groups) was double that of their mothers (27.1%, ≥ 5 of 10 food groups). Dietary adequacy amongst women increased across successive data collection periods, from 18.4% in November 2014 to 39.4% in May 2016. Similar increases for breastfed (21.3% to 66.7%) and non-breastfed children (34.7% to 68.6%) were noted. 

**Figure 5:**
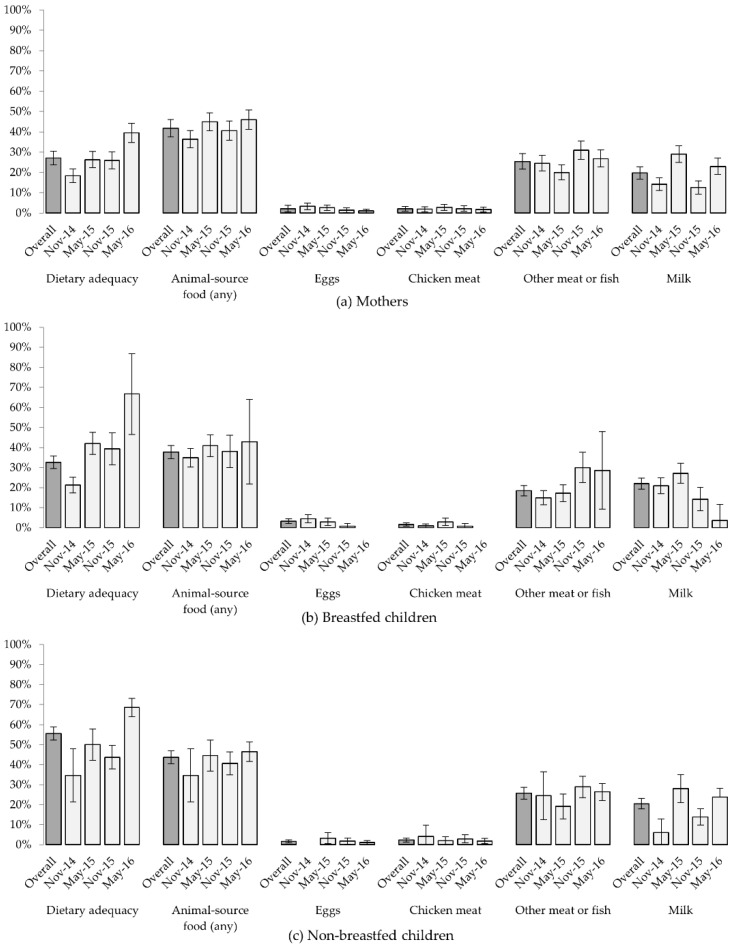
Percentage of (**a**) mothers, (**b**) breastfed children, and (**c**) non-breastfed children with adequate diets (according to MDD-W and IYCMDD, respectively) and consuming animal-source foods, based on six-monthly 24-h food recall, overall and for each six-monthly data collection. 95% confidence intervals are shown. Low numbers of non-breastfed children during early data collection periods, and of breastfed children later in this longitudinal study, have resulted in wide confidence intervals for some percentages reported.

Consumption of eggs and chicken meat was very uncommon (2.1% mothers for each item, across all records), and markedly less common than other forms of meat or fish (25.4%) and milk (19.7%). Milk consumption varied prominently between data collection periods. With the exception of the small number of breastfed children in May 2016 (*n* = 21), records indicate milk to be more commonly consumed during May than in November (Figure 5). 

#### 3.3.2. Univariable and Multivariable Models

Unconditional associations between predictor variables of interest and dietary outcomes are included in Appendix B (Table A2). Several measures of chicken ownership were significantly associated with positive dietary outcomes in univariable models; however, a majority of these were not significant in multivariable models (Table 3). For example, despite positive unconditional associations (*p* < 0.001) between the number of chickens owned by a household and DD scores for mothers and breastfed children, no significant associations were identified when controlling for household wealth. One notable exception to this pattern was the consumption of ASF by women, which was positively associated with both the number of chickens (*p* = 0.009) and cattle (*p* = 0.005) owned, but not with measures of wealth based on other assets (*p* = 0.190). 

In final multivariable models (Table 3), across all participant groups, no significant differences in dietary outcomes were detected between categories of chicken-keeping and non-chicken-keeping households. In two models, one assessing the probability of dietary adequacy for mothers and one evaluating DD scores amongst breastfed children, positive associations were identified with ownership of greater than the median number of chickens (i.e., more than four birds). The number of chickens owned by a household, tested as a continuous variable, was also positively associated with DD scores for non-breastfed children (*p* = 0.038), and chicken consumption by breastfed children (*p =* 0.016), and suggested to be positively associated with chicken consumption by mothers (*p* = 0.053). 

In all three participant groups, egg consumption was not associated with chicken ownership, but rather with the adjusted HDAI. Despite infrequent egg consumption across the study population, mothers in the highest wealth quintile were identified to have 16.5 times greater odds of consuming eggs than those in the lowest quintile (0.038 probability vs. 0.002), and breastfed children in the highest quintile to have 17.9 times greater odds than those in the lowest quintile (0.053 vs. 0.003). In contrast to the lack of association between chicken ownership and egg consumption, cattle numbers were a strong predictor of milk consumption for all groups (*p* < 0.001), allowing for the influence of season and language group (with households identifying as Sukuma significantly more likely to consume milk (*p* < 0.001 for mothers and non-breastfed children, *p* = 0.002 for breastfed children)). 

For all groups, significant variation in DD scores, dietary adequacy and milk consumption were seen between months of dietary assessments. Diets were more diverse and more likely to meet thresholds for dietary adequacy, and milk more likely to be consumed in May (shortly after the end of the rain season) than in November. No significant associations were detected between household size or mothers’ formal education and diets.

Both women and breastfed children were more likely to consume eggs in a female-headed household compared with a male-headed one (*p* < 0.001 and *p* = 0.032, respectively). While model-based predictions were low across all groups, breastfed children in female-headed households were determined to have 2.6 times greater odds of consuming eggs, compared to those in male-headed households (0.041 probability vs. 0.016). Being part of a female-headed household was also suggested to be associated with higher DD scores for women (mean of 3.90 vs. 3.74 for male-headed households, *p* = 0.068), but a lower likelihood of consuming milk (*p* = 0.011), controlling for variation in cattle ownership and language group. 

Increasing child age was significantly associated with higher DD scores (*p* < 0.001) and a higher probability of an adequately diverse diet (*p* < 0.001) for breastfed children, but not for non-breastfed children. Across the range of dietary outcomes evaluated, gender-based differences were only evident amongst the non-breastfed group. Male children were significantly more likely to meet the cut-off for dietary adequacy (*p* = 0.045) and to consume ASF (*p* = 0.014), and suggested to have higher DD scores (*p* = 0.066), compared to female children. 

### 3.4. Height-for-Age and Diarrhea in Children

#### 3.4.1. Prevalence of Stunting

Summaries of anthropometric data were disaggregated by ward and by time period (Table 4). The prevalence of stunting increased from 36.8% to 49.5% over the first three data collection periods in Sanza Ward, reducing to 39.8% at the time of the final data collection. In Majiri Ward, where the mean age of children was lower at enrolment (7.6 mo vs. 9.9 mo; *p* < 0.001), a continuing increase in the prevalence of stunting was seen over successive data periods, from 28.3% to 53.0%. 

#### 3.4.2. Univariable and Multivariable Models

As for dietary outcomes, univariable models were first used to test unconditional associations between demographic, socioeconomic and livestock-associated variables and (a) HAZ, (b) probability of stunting, and (c) diarrhea in children. Variables relating to the consumption of ASF by children during the day prior to anthropometry were also included. These short-term dietary indicators are unlikely to be associated with long-term outcomes such as HAZ; however, the potential for patterns to be identified at a population level was considered adequate to warrant their consideration. Unconditional associations based on univariable analysis are included in Appendix B (Table A3).

Children’s age, gender, diarrhea frequency, household language group and NLAI quintiles were significantly associated with HAZ within univariable linear mixed models (Table A3), and remained significant at the 5% level in a multivariable model (Table 5). Height-for-age Z-scores were negatively associated with increasing child age, the male gender, more frequent diarrhea, language groups other than Sukuma, and lower NLAI scores. Of the livestock and dietary variables identified as being significantly or suggestively associated with HAZ in univariable models, none were significant in multivariable models. These included the number of chickens owned by a household (*p* = 0.960 in multivariable model), livestock ownership as a bivariate categorical variable (*p* = 0.409), and children’s consumption of milk (*p* = 0.802) or meat or fish (*p* = 0.236) during the previous day. 

**Figure 6:**
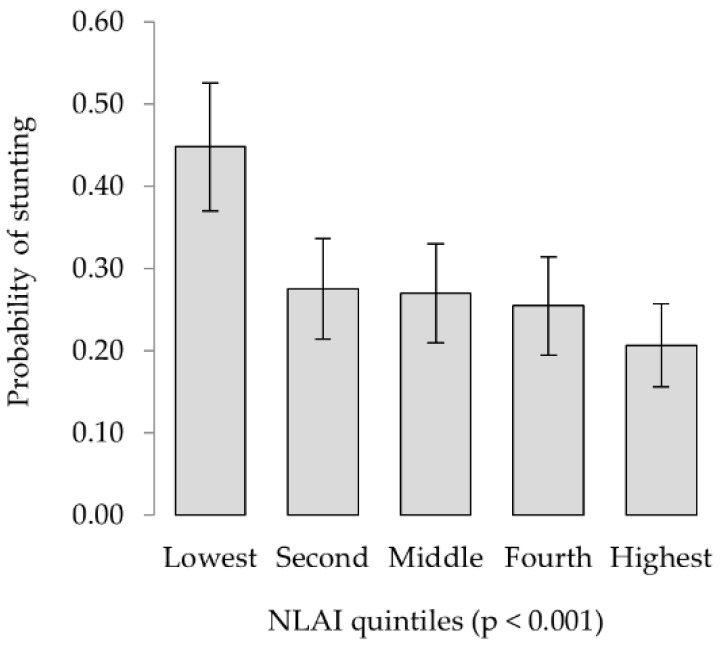
The non-livestock asset index was significantly associated with the probability of child stunting, with a significantly higher likelihood identified amongst the lowest wealth quintile (*p* < 0.001).

Similar associations were found when evaluating stunting as a binary outcome, with a higher probability of stunting linked to increasing age, male children, language groups other than Sukuma and lower asset scores, and no significant associations with any livestock or dietary variables. Univariable analysis indicated a suggestive association with the HDAI (livestock and non-livestock assets), but it was the NLAI (non-livestock assets only) which was highly significantly associated (*p* < 0.001) with the probability of stunting in both uni- and multivariable models (Table A3 and Table 5). Particularly poor outcomes were associated with the lowest NLAI quintile, including 2.1 times greater odds of stunting compared to children in the middle quintile, and 3.1 times greater odds than those in the highest quintile (Figure 6).

**Figure 7:**
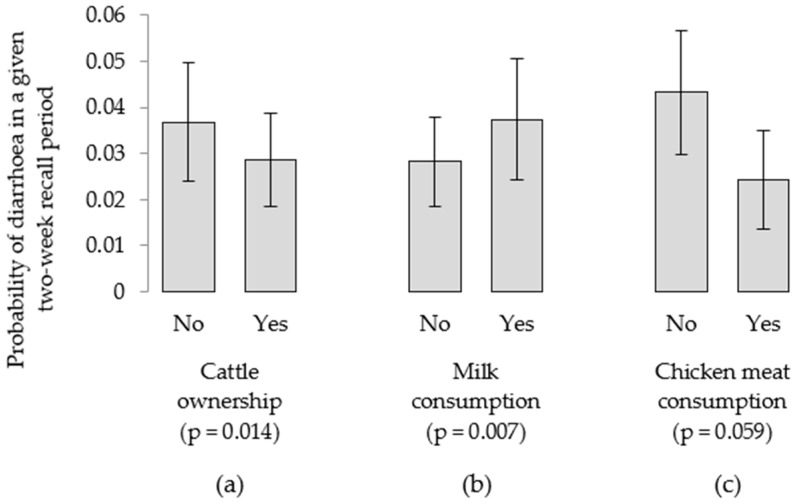
Probability of child diarrhea being reported in a given two-week period, according to (**a**) household cattle ownership, and (**b**) children’s consumption of milk or (**c**) chicken meat the previous day.

Multivariable models revealed a significantly lower likelihood of diarrhea with increasing child age (*p* < 0.001). Ownership of cattle was associated with a small but significant reduction in the probability of child diarrhea (*p* = 0.021), with a 0.036 probability of diarrhea in a given fortnight for a child in a cattle-owning household, compared to a 0.045 probability in a household without cattle (Figure 7a). Controlling for child age and cattle ownership, however, milk consumption was linked to an increased probability of diarrhea (*p* = 0.007), based on a single 24-h dietary assessment conducted in each six-month period of diarrhea records (Figure 7b). There was also a suggestive association (*p* = 0.059) between consumption of chicken meat and a lower probability of child diarrhea. When variables related to chicken-keeping were tested in the same model, no significant difference in the number of records of child diarrhea was evident according to chicken ownership (*p* = 0.305), chicken flock size (*p* = 0.498), or the practice of keeping chickens inside the home overnight (*p* = 0.550).

## 4. Discussion

### 4.1. Seasonal and Temporal Variation in Diets

The opportunity to detect seasonal influences on diets has been limited by the two-year span of data. Seasonality is a key determinant of dietary adequacy in many vulnerable rural households, yet is sometimes overlooked in dietary assessments [52]. In this setting, where rain-fed agriculture is the predominant source of income and food, there is a risk of food shortage between the depletion of one year’s cereal stocks and the following year’s harvest. Poultry mortality due to disease outbreaks, with signs consistent with Newcastle disease, are most common between July and November in this area [53] and may also contribute to seasonal fluctuations in the availability of food resources and assets. 

Of the months when dietary assessments were conducted, May would typically be a time of greater food availability (due to recently-harvested crops and associated income, and a lower risk of chicken mortality) and November a time of food scarcity for less resilient households (when crop supplies have been depleted, income-earning opportunities are scarce, and losses due to Newcastle disease likely amongst unvaccinated chicken flocks). However, daily rainfall records indicate the 2014–2015 rain season to have been particularly poor, with a total rainfall of 447 mm (30 days) in Sanza Ward and 275 mm (21 days) in Majiri Ward, substantially lower than the long-term mean annual rainfall of 624 mm (49 days) for the district [39]. A poor harvest is likely to have resulted in lower dietary diversity in May 2015 than would be common at this time of year.

While graphical summaries suggest increasing levels of dietary adequacy over time (Figure 5), this is likely to reflect diversification of children’s diets with age (*p* < 0.001). Increasing age was associated with higher DD scores and a higher probability of dietary adequacy amongst breastfed children, but this did not continue beyond the point of weaning. Multivariable models, controlling for children’s age, confirm the month of assessment to be highly significantly associated with dietary diversity scores, dietary adequacy and milk consumption for children and their mothers (Table 3) reflecting the expected improved diets in May compared to November. 

### 4.2. Chicken-Keeping and Diets

Egg consumption was documented in only 3.2% of all records for breastfed children, 2.1% for women and 1.6% for non-breastfed children—even lower than the 5.2% reported amongst children under two years in the most recent national dietary assessment [54]. Associations between egg consumption and household wealth, but not chicken-keeping, suggest it is the financial capacity to purchase eggs through markets—rather than access to eggs from their own hens—which determined consumption frequency during this period. This supports findings elsewhere in African communities accustomed to high levels of mortality in scavenging chicken flocks, where households prioritize the hatching of eggs for replacement stock and retention of chickens for sale in times over home consumption [55,56]. Previous qualitative work in the study setting found no currently-held beliefs or cultural constraints to explain provide other explanations for infrequent egg consumption [57].

A significant increase in the likelihood of breastfed children consuming chicken meat with increasing chicken flock size, and a suggestive increase in the case of women, is encouraging. It is likely that initial unconditional associations between larger chicken flocks and improved dietary outcomes (such as higher DD scores) are due to flock size acting as a proxy for socioeconomic status. The significance of this association disappeared in a majority of the multivariable models, as alternative measures of household wealth were included. One exception to this was the finding of a greater likelihood of women consuming ASF with increasing numbers of chickens and cattle, but not with wealth estimation based on other assets. 

In the case of cattle, this effect is likely to reflect access to milk (as shown in the specific model for milk consumption). Based on low levels of chicken meat and egg consumption, however, alternative mechanisms through which women’s ASF consumption might benefit from chicken ownership should be considered. Motivations for chicken-keeping in resource-poor settings include opportunities to store wealth in a form which is readily-accessible to meet immediate household needs, including education and medical expenses, and items such as cooking oil, soap or clothing for children [55]. It may also be the case that, even if poultry products are rarely eaten at home, the sale of chickens may facilitate access to other forms of ASFs—fresh or dried fish, other forms of meat, or milk—through local markets.

### 4.3. Livestock and Child Growth

For 39.6% of study participants, livestock constituted more than three-quarters of their household’s wealth, yet none of the measures of livestock-keeping tested were associated with children’s height-for-age. It was notable that a reduced probability of stunting was associated with the non-livestock asset index, but not with the combined measure of livestock and non-livestock wealth. The concept of the “livestock ladder” emphasizes the role of livestock-keeping in poverty alleviation [32], and posits that increasing economic benefits are derived from keeping larger livestock species [58]. Since no negative associations between animal ownership and stunting were found, the observed dilution effect of including livestock in estimations of wealth indicate that these suggested economic benefits have not translated to improved growth outcomes for children in this setting during the study period. 

Livestock-keeping in resource-poor settings is often not oriented towards production for market [6]. Animals represent financial savings and, particularly in the case of cattle, social status. Animal numbers may increase through the retention of offspring, adding to household wealth, but such assets may contribute little to children’s diets and growth. In this study, the non-livestock asset index was based on items such as mobile phones, radios, and bicycles, which demonstrate past expenditure and may serve as a better proxy for households’ purchasing behavior. A demonstrated association between this index and lower levels of stunting may reflect the utilization of economic resources to address underlying determinants of nutritional status, through nutritious food purchases, medical expenses or an improved home environment.

This argument is countered by the finding of increased milk consumption by children with increasing numbers of cattle, signifying greater access to a food item which has been shown to improve linear growth amongst stunted children [59]. Quantitative dietary assessments were not conducted, but the volume of milk consumed is likely to be relatively low in this setting, particularly in times of limited feed and water availability for cattle, and perhaps insufficient to influence growth. It is also possible that the lack of significance of cattle ownership for child height-for-age may signify the net effect of nutritional benefits of increased milk consumption being counteracted by undocumented adverse impacts, such as EED. 

### 4.4. Livestock and Child Diarrhea

Twice-monthly records of child diarrhea and information on chicken numbers and housing location have provided an opportunity to explore associations more deeply than has been possible through analyses based on multi-purpose national surveys [24,27]. Keeping chickens inside human dwellings overnight is a common practice in this project setting, reported by over two-thirds (64.2%) of chicken-keeping households and more than a third (35.3%) of all those with a child enrolled in this study. Chicken houses may be damaged or destroyed by heavy rainfall during the wet season, and there is often little incentive to construct or repair these facilities when rates of chicken mortality are high and poultry-keeping is not a priority livelihood activity. 

When relationships were tested between longitudinal child diarrhea records and households’ ownership of chickens, chicken flock size and the location of overnight chicken housing, no significant associations were identified. In the case of cattle, ownership was associated with a small but significantly lower probability of diarrhea; however, milk consumption the previous day was linked to an increased incidence of child diarrhea. The potential for milk contamination with zoonotic pathogens or aflatoxins [60,61,62] to have negatively affected linear growth [24], cannot be excluded and, although highly speculative within this study, warrants closer investigation. 

In a setting where infants and young children commonly accompany their mother to agricultural plots or are left in the care of older siblings, neighbors or relatives, opportunities for exposure to poultry feces within the homestead or broader environment are frequent, and not restricted to those households keeping chickens. It is therefore possible that free-ranging village chickens present an opportunity for “community-wide” adverse child health impacts which have not been detected in this study. 

### 4.5. Gender and Nutrition

Much attention is given to the role of women as key mediators of agriculture-nutrition linkages. Consideration of the gender of the “household head” has attracted some criticism, because of the ambiguity in defining this role, its implication of a hierarchical relationship, and the common gender bias whereby the oldest male household member is taken as the “head” [63,64]. Assumptions about vulnerability based on this concept have also been questioned, with female-headed households at very low levels of income in Kenya shown to adopt successful coping strategies and achieve higher weight-for-age of preschool-aged children than wealthier male- and female-headed households [65]. In the present study, both women and their breastfed children were more likely to consume eggs in households identifying as female-headed. This may reflect women’s greater autonomy in decision-making or differing priorities for resource allocation, compared to male-headed households. 

There were no significant gender-based differences in breastfed children’s diets; however, amongst non-breastfed children, boys were more likely than girls to receive an adequately diverse diet and to consume ASF. Preferential treatment of sons over daughters has been commonly reported in ethnographic and demographic research [66]. Studies in South Asia indicate that biased food allocation which favors male children is more apparent amongst higher wealth groups, and least evident amongst the very poor [67]. A puzzling situation exists in this study where male children were associated with some improved dietary indicators, and also with lower HAZ and a higher probability of stunting, compared with female children. 

Gender-based differences in stunting, with poorer growth amongst boys, have been reported in several studies in sub-Saharan Africa [68,69,70]. The cause of this disparity is not well understood, particularly given that cases of female-biased parental investment have been rarely documented [66]. A meta-analysis of 60 national surveys from sub-Saharan Africa found no evidence of gender-based differences in breastfeeding duration or maternal health-seeking behaviors, based on rates of vaccination and the use of oral rehydration therapy [71]. The authors contended that biological differences (a concept not further elaborated in the review) may account for gender-based inequalities in the prevalence of stunting. The current study’s finding of improved growth outcomes for girls, despite evidence of dietary practices which favor male children, appears to support this theory.

### 4.6. Limitations of This Study

Low and abnormally-timed rainfall over two consecutive rain seasons resulted in an unforeseen level of mobility amongst participating households, with a drastically reduced harvest prompting some to relocate outside the area to pursue alternative livelihood strategies. This contributed to an attrition rate of 16.9% over the study period. The potential for attrition-related bias, in which the characteristics of those lost to follow-up is associated with the outcome of interest, has been suggested as a consideration for losses of between 5–20% of participants [72]. With increasing weather variability in the future, studies of populations reliant on rain-fed agriculture should adjust sample size calculations in recognition of the potential for increased participant drop-out, and consider the implications of more vulnerable, resource-poor segments of a population being lost from the study.

A further limitation of this study has been the reliance on self-report of diarrhea. Recall periods of longer than two days have been associated with under-reporting of diarrhea, particularly milder episodes and amongst older children [73]. Given the regularity of data collection over an extended period in this study, the influence of respondent or enumerator fatigue is possible. However, the potential biases of inaccurate recall and recording are considered to be systemic and unlikely to influence associations between diarrhea frequency and livestock ownership. The use of well-regarded community members for ongoing data collection and monthly field visits by the Tanzanian research team are hoped to have contributed to maintaining positive relationships with study participants, and avoiding the pitfalls of “parachute research” [74]. 

## 5. Conclusions

The potential for livestock-keeping to sustainably improve food and nutrition security has long been recognized, yet (as for other agricultural interventions [75]) impact has been notoriously difficult to demonstrate. To address the key question of whether chicken-keeping has an impact on the growth of young children in this setting, use of height-for-age as an outcome measure sought to encompass multiple dimensions of children’s health, development, and environment. On balance, there were no significant associations between HAZ and chicken ownership, when the latter was defined (a) as a dichotomous categorical variable, (b) using a threshold approach, (c) in terms of chicken flock size, and (d) accounting for overnight housing location. While the potential for community-wide effects related to free-roaming poultry cannot be ruled out, this study importantly (and of substantial practical significance if confirmed) found no indication of a heightened risk of stunting or greater frequency of diarrhea being associated with chicken ownership or the practice of keeping chickens within human dwellings overnight. 

Livestock ownership was closely linked to socioeconomic status in this population; however, controlling for other forms of wealth, analyses have detected no influence of any categories of livestock on children’s growth. This raises questions as to whether the economic benefits of climbing the “livestock ladder” translate into nutritional benefits for children, yet methodological constraints and challenging weather patterns during the study period must be acknowledged. Alongside two years of poor rainfall and dramatic crop losses, it is likely that the multiple and diverse household needs met by livestock-keeping have not included adequate ASF consumption or dietary diversification to influence children’s growth during this time.

Chicken ownership was significantly associated with more frequent consumption of ASF by women and chicken meat by young children. Although chicken-keeping was not a significant determinant of additional dietary outcomes, contributions to socioeconomic status and resilience should not be overlooked. Improved water, sanitation and hygiene practices are central to efforts to reduce stunting; however, there is a need for careful consideration before warning against livestock ownership by vulnerable households or proposing substantial changes in livestock management practices. For example, confining chickens to enclosures will increase production costs and labor inputs, particularly for women, and will reduce their accessibility to poor families. 

As efforts to support rural households to enhance dietary quality continue, questions about the complex and multiple linkages between livestock and human nutrition will endure. In this study, findings of no net benefit of cattle ownership on child growth despite a strong positive association with milk consumption warrant further investigation using mixed-methods approaches. While contributions of chicken-keeping to diets were limited during the study period, it is encouraging that no adverse impacts have been found. Integrated, multi-sectoral approaches will be central to increasing chicken flock size in vulnerable households, building resilience in the face of increasing weather variability, and developing nutritional messaging to promote home consumption of chicken meat and eggs, in order to harness the nutritional potential of chicken-keeping in support of children’s growth and development.

## Figures and Tables

**Table 1:** Indicators to represent household socioeconomic status in this study.

Indicator	Definition	Use
Household Domestic Asset Index (HDAI)	A weighted sum of material and livestock assets	To characterize households’ socioeconomic status in descriptive summaries of the study population.
HDAI excluding cattle and chickens	A weighted sum of material and selected livestock assets (sheep, goats, donkeys, pigs)	To control for variation in socioeconomic status in models for maternal and child diets, where the influence of cattle and chicken ownership was tested separately.
Non-Livestock Asset Index (NLAI)	A weighted sum of material assets only	To control for variation in socioeconomic status in models for child stunting and diarrhea, where multiple livestock-associated variables were tested separately.

**Table 2:** Overview of study population according to baseline questionnaire responses, by ward and overall.

Location	Sanza Ward	Majiri Ward	Overall
Baseline data collection	May 2014	Nov 2014	
Enrolled households (*n*)	229	274	503
Sex of child, female (%)	55.5	47.4	51.1
Child age in months			
Mean (SD)	9.9 (6.1) ^a^	7.6 (4.3) ^a^	8.6 (5.3)
Range	1.2–28.1	0.6–22.5	0.6–28.1
Maternal age in years			
Mean (SD)	28.5 (7.5) ^a^	26.8 (7.5) ^a^	27.7 (7.6)
Range	15–50	13–54	13–54
Age unknown (%)	9.2 ^b^	23.7 ^b^	17.1
Maternal education (%)			
No formal education	22.7 ^b^	40.5 ^b^	32.4
Some primary school	68.6 ^b^	56.6 ^b^	62.0
Some secondary school	5.7 ^b^	1.5 ^b^	3.4
Unspecified level	3.1	1.5	2.2
Primary language of household (%)			
*Kigogo*	78.2 ^b^	74.8 ^b^	76.3
*Kisukuma*	6.1 ^b^	14.6 ^b^	10.7
Other	4.4	2.6	3.4
Unspecified	11.4 ^b^	8.0 ^b^	9.5
Parents of same language group (%)	92.1	95.6	94.1
Number of household members			
Mean (SD)	5.6 (2.0)	5.5 (2.3)	5.5 (2.2)
Range	2–16	2–21	2–21
Female-headed households (%)	30.2 ^b^	16.4 ^b^	22.7
Socioeconomic status, median (IQR)			
Non-livestock and livestock assets, HDAI	12 (5–51)	26 (7–115)	19 (7–76)
Non-livestock assets only, NLAI	7 (3–11)	9 (3–13)	9 (3–12)
Livestock ownership (%)			
Cattle	26.7 ^b^	36.2 ^b^	31.8
Sheep or goats	27.1 ^b^	47.8 ^b^	38.3
Chickens	51.1 ^b^	42.1 ^b^	46.3
Number of livestock, median (IQR) ^c^			
Cattle	4 (2–17) ^a^	10 (4–20) ^a^	7 (4–20)
Sheep or goats	14 (7–20)	12 (5–25)	12 (6–24)
Chickens	7 (2–13)	8 (5–13)	8 (4–13)
Improved water source (%)	2.6	2.2	4.9
Improved toilet facilities (%)	3.1 ^b^	0.4 ^b^	1.6

Significant differences between wards (*p* < 0.05), as determined by ^a^ *t*-tests and ^b^ chi-square tests. ^c^ Amongst households owning livestock, by category.

**Table 3:** Multivariable models ^a^ for maternal and child dietary adequacy, dietary diversity and ASF consumption, showing *p*-values and the direction of significant (*p* < 0.05) and suggestive (0.05 ≤ *p* < 0.1) associations. Grey shading indicates significant and suggestive associations in univariable models (Table A2), and “NS” denotes non-significant associations in final multivariable models.

Predictor Variables	Dietary Adequacy	DD Score ^b^	ASF Consumption			
			Any	Chicken	Egg	Milk
*(a) Mothers*						
Month of dietary assessment, May	<0.001 (+)	<0.001 (+)	0.006 (+)	NS	NS	<0.001 (+)
Maternal age	NS	0.067 (−)	NS	NS	0.092 (−)	NS
Maternal formal education, yes	NS	NS	NS	NS	NS	NS
Breastfeeding, yes	NS	NS	NS	NS	NS	NS
Sex of household head, female	NS	0.068 (+)	NS	NS	<0.001 (+)	0.014 (−)
Number of household members	NS	NS	NS	NS	NS	NS
Language group, Sukuma	NS	NS	0.032 (+)	NS	NS	<0.001 (+)
Household domestic asset index^c,d^	0.002 (+)	< 0.001 (+)	NS	0.058 (+)	0.005 (+)	NS
Chickens owned, yes	NS	NS	NS	NS	NS	NS
Chickens, above median number	0.023 (+) ^e^	NS	NS	NS	NS	NS
Chickens, number owned ^c^	0.032 (+) ^e^	NS	0.009 (+)	0.053 (+)	NS	NS
Cattle owned, yes	NS	NS	NS	NS	NS	NS
Cattle, above median number	NS	NS	NS	NS	NS	NS
Cattle, number owned ^c^	NS	NS	0.005 (+)	NS	NS	<0.001 (+)
*(b) Breastfed children*						
Month of dietary assessment, May	0.002 (+)	0.057 (+)	NS	NS	NS	0.028 (+)
Child age	<0.001 (+)	<0.001 (+)	NS	NS	NS	NS
Sex of child, female	NS	NS	NS	NS	NS	NS
Maternal formal education, yes	NS	NS	NS	NS	NS	NS
Sex of household head, female	NS	NS	NS	NS	0.032 (+)	NS
Number of household members	NS	NS	NS	NS	NS	NS
Language group, Sukuma	0.046 (+)	NS	0.014 (+)	NS	NS	0.002 (+)
Household domestic asset index ^c,d^	NS	0.002 (+)	< 0.001 (+)	NS	<0.001 (+)	NS
Chickens owned, yes	NS	NS	NS	NS	NS	NS
Chickens, above median number	NS	0.039 (+)	NS	NS	NS	NS
Chickens, number owned ^c^	NS	NS	0.083 (+)	0.016 (+)	NS	NS
Cattle owned, yes	NS	NS	NS	NS	NS	0.010 (+) ^f^
Cattle, above median number	NS	NS	NS	NS	NS	<0.001 (+) ^f^
Cattle, number owned ^c^	<0.001 (+)	NS	NS	NS	NS	<0.001 (+) ^f^
*(c) Non-breastfed children*						
Month of dietary assessment, May	<0.001 (+)	<0.001 (+)	0.003 (+)	NS	NS	<0.001 (+)
Child age	NS	NS	NS	NS	NS	NS
Sex of child, female	0.045 (−)	0.066 (−)	0.014 (−)	NS	NS	NS
Maternal formal education, yes	NS	NS	NS	NS	NS	NS
Sex of household head, female	NS	NS	NS	NS	NS	NS
Number of household members	NS	NS	NS	0.080 (+)	0.059 (−)	NS
Language group, Sukuma	NS	NS	0.002 (+)	NS	NS	<0.001 (+)
Household domestic asset index ^c,d^	NS	NS	NS	NS	0.023 (+)	NS
Chickens owned, yes	NS	NS	NS	NS	NS	NS
Chickens, above median number	NS	NS	NS	NS	NS	NS
Chickens, number owned ^c^	NS	0.038 (+)	NS	NS	NS	NS
Cattle owned, yes	NS	NS	NS	NS	NS	0.003 (+)
Cattle, above median number	NS	NS	NS	NS	NS	0.050 (+)
Cattle, number owned ^c^	NS	NS	NS	NS	NS	<0.001 (+)

^a^ Generalized linear mixed models using binomial distribution, allowing for geographic clustering and longitudinal data; ^b^ Binomial totals of 10 for women (MDD-W) and 7 for children (IYCMDD); ^c^ log-transformed variables used to minimize excessive influence of large numbers; ^d^ Cattle and chickens excluded from HDAI, evaluated as separate predictor variables; ^e^ Two alternative models were constructed, one using the number of chickens owned and one using above-/below-median flock size, each together with the month of dietary assessment and HDAI (for which *p-*values remained unchanged); ^f^ Three alternative models were constructed, one using cattle ownership as a dichotomous variable, one using above-/below-median cattle herd size and one using the number of cattle owned, each with the month of dietary assessment and language group (for which *p-*values remained unchanged).

**Table 4:** Overview of height-for-age Z-scores (HAZ) and the percentage of stunting amongst enrolled children, by ward and by data collection period.

	Mean HAZ (SD)	% Stunting	*n*
Sanza Ward			
May 2014	−1.52 (1.13)	36.8	220
Nov 2014	−1.63 (1.18)	34.5	200
May 2015	−2.02 (1.14)	49.5	202
Nov 2015	−1.98 (1.05)	48.2	191
May 2016	−1.77 (1.05)	39.8	201
Majiri Ward			
Nov 2014	−1.45 (1.21)	28.3	272
May 2015	−1.86 (1.05)	41.4	261
Nov 2015	−1.99 (0.98)	49.6	234
May 2016	−2.16 (1.00)	53.0	217

**Table 5:** Multivariable models for height-for-age Z-scores (HAZ) ^a^, probability of stunting (HAZ < −2) ^b^ and diarrhea frequency ^b^ in children, showing *p*-values and the direction of significant (*p* < 0.05) and suggestive (0.05 ≤ *p* < 0.1) associations. Grey shading indicates significant and suggestive associations in univariable models (Table A3), and “NS” denotes non-significant associations in final multivariable models.

Predictor Variables	HAZ	Stunting	Diarrhea
Child age	<0.001 (−)	<0.001 (+)	<0.001 (−)
Sex of child, female	0.022 (+)	0.002 (−)	NS
Diarrhea frequency	<0.001 (−)	NS	N/A
Height-for-age Z-score	N/A	N/A	NS
Month of data collection, May ^c^	NS	NS	NS
Sex of household head, female	NS	NS	NS
Number of household members	NS	NS	NS
Maternal formal education, yes	NS	NS	NS
Household language group, Sukuma	<0.001 (+)	0.002 (−)	NS
Improved water source	NS	NS	NS
Improved toilet facility	NS	NS	NS
Household domestic asset index			
Livestock and non-livestock assets ^d^	NS	NS	NS
Non-livestock assets only ^d^	NS	<0.001 (−) ^f^	NS
Non-livestock assets only, quintiles	0.009 (+)	<0.001 (−) ^f^	NS
Livestock			
Livestock owned, yes	NS	NS	NS
“Livestock ladder” ^e^	NS	NS	NS
Chickens owned, yes	NS	NS	NS
Chickens, above median	NS	NS	NS
Chickens, number owned ^d^	NS	NS	NS
Chickens, location of overnight housing	NS	NS	NS
Sheep or goats owned, yes	NS	NS	NS
Sheep or goats, above median	NS	NS	NS
Sheep or goats, number owned ^d^	NS	NS	NS
Cattle owned, yes	NS	NS	0.014 (−)
Cattle, above median	NS	NS	NS
Cattle, number owned ^d^	NS	NS	NS
Children’s diet, previous 24 h			
ASF consumption, yes	NS	NS	NS
Chicken meat consumption, yes	NS	NS	0.059 (−)
Other meat or fish consumption, yes	NS	NS	NS
Egg consumption, yes	NS	NS	NS
Milk consumption, yes	NS	NS	0.007 (+)

^a^ Linear mixed models, allowing for geographic clustering and longitudinal data; ^a^ Generalized linear mixed models using binomial distribution, allowing for geographic clustering and longitudinal data; ^c^ log-transformed variables used to minimize excessive influence of large numbers; ^d^ Two data collection months: May and November. Rainfall typically occurs between November and April in this area.; ^e^ Levels of livestock ownership (the “livestock ladder”): (1) none, (2) chickens only, (3) small ruminants +/− chickens, no cattle, (4) cattle +/− chickens and small ruminants; ^f^ Two alternative models were constructed, one using the NLAI as a continuous variable and one using the NLAI as quintiles, each together with the age and sex of child and household language group (*p-*values for these latter variables remained unchanged).

## Abbreviations

EED	environmental enteric dysfunction
HAZ	height-for-age
mo	months of age
DD	dietary diversity
MDD-W	Minimum Dietary Diversity for Women of Reproductive Age
IYCMDD	Infant and Young Child Minimum Dietary Diversity
HDAI	Household Domestic Asset Index
NLAI	Non-Livestock Asset Index
LMIC	low- to middle-income country

## Appendix A

**Table A1:** Descriptions for predictor and outcome variables evaluated.

	Variable(s)	Description
Child	Age	From maternal recall; verified against clinic health records if available
	Gender	Male or female
	HAZ	From six-monthly length or height measurements [41]
	Diarrhea score	From ratio of positive records of diarrhea, as reported by child’s mother or primary caretaker, to total number of records per child
Mother and child	Dietary diversity	Number of food groups reported as consumed by mothers (of 10 groups [40]) and children (of 7 groups [43]) during day or night prior to interview
	Dietary adequacy	Dichotomous variable, based on minimum cut-offs for women (≥5 of 10 food groups [40]) and children (≥4 of 7 groups [43])
	ASF consumption	Dichotomous variables, according to reports of food consumed during the day or night prior to interview. Categories considered are eggs, chicken meat, other meat and fish, and milk.
Household demographics	Maternal age	Based on mother’s recall, or calculated from self-reported date of birth if age unknown
	Maternal formal education	Dichotomous variable for no formal education vs. some level of primary or secondary school education
	Household size	Total number of household members, with a household defined as people living together and sharing food at least three days of each week for the previous six months [45].
	Household head	Male or female, as reported by questionnaire respondent (any household member > 16 years of age; intended even numbers of men and women).
	Household language group	Dichotomous variable distinguishing the *Kisukuma* language group from other groups; determined from the first language (“mother tongue”) of both parents of enrolled children and the gender of the household head.
Water and sanitation	Water source	Categorized as improved or unimproved [49]
	Toilet facility	Categorized as improved or unimproved [49]
Socioeconomic status	Household Domestic Assets Index (HDAI)	Weighted sum of livestock and non-livestock assets, according to their relative value using a system developed for use in sub-Saharan Africa [46] *For dietary models:* Chickens and cattle were excluded from the HDAI, to test their association with dietary outcomes separately.
	Non-Livestock Asset Index (NLAI)	Weighted sum of non-livestock assets only [46] *For stunting and diarrhea models:* NLAI used rather than HDAI, to control for non-livestock wealth while testing a range of livestock variables separately as predictors of growth and diarrhea in children.
Livestock ownership	Livestock ownership	Dichotomous variable (yes / no) for owning any form of livestock
	Cattle ownership	Dichotomous variable (yes / no) for owning cattle Dichotomous variable for owning more or less than the median number of cattle in this population (i.e., > 7 vs. ≤ 7) Number of cattle owned.
	Small ruminant ownership	Dichotomous variable (yes / no) for owning sheep or goats Dichotomous variable for owning more or less than the median number of sheep or goats in this population (i.e., > 11 vs. ≤ 11) Number of sheep or goats owned
	Chicken ownership	*For dietary models*, based on chicken ownership during the month of dietary assessment. *For stunting and diarrhea models*, based on ownership over the six-month periods preceding each round of anthropometry. In both cases: Dichotomous variable (yes / no) for owning chickens Dichotomous variable for owning more or less than the median number of sheep or goats in this population (i.e., >11 vs. ≤11) Number of sheep or goats owned

## Appendix B

**Table A2:** Univariable models ^a^ evaluating the significance of predictor variables for maternal and child dietary adequacy, dietary diversity and ASF consumption, showing *p*-values and the direction of associations (+/−). Grey shading indicates all suggestive associations (*p* < 0.1).

Predictor Variables	Dietary Adequacy	DD Score ^b^	ASF Consumption			
			Any	Chicken	Egg	Milk
*(a) Mothers*						
Month of dietary assessment, May	<0.001 (+)	<0.001 (+)	<0.001(+)	0.694	0.343	<0.001 (+)
Maternal age	0.393	0.299	0.043 (−)	0.976	0.423	0.157
Maternal formal education, yes	0.090 (+)	0.095 (+)	0.939	0.465	0.959	0.239
Breastfeeding, yes	<0.001 (−)	< 0.001 (−)	0.022 (−)	0.887	0.018 (+)	0.377
Sex of household head, female	0.502	0.725	0.284	0.428	0.015 (+)	0.004 (−)
Number of household members	0.815	0.944	0.864	0.213	0.689	0.016 (+)
Language group, Sukuma	0.484	0.118	0.001 (+)	0.120	0.486	<0.001 (+)
Household domestic asset index ^c,d^	<0.001 (+)	<0.001 (+)	<0.001 (+)	0.024 (+)	0.005 (+)	<0.001 (+)
Chickens owned, yes	0.004 (+)	0.003 (+)	0.003 (+)	0.036 (+)	0.685	0.011 (+)
Chickens, above median number	<0.001 (+)	0.009 (+)	0.011 (+)	0.463	0.148	0.003 (+)
Chickens, number owned ^c^	<0.001 (+)	<0.001 (+)	<0.001 (+)	0.013 (+)	0.159	<0.001 (+)
Cattle owned, yes	0.003 (+)	0.002 (+)	<0.001 (+)	0.251	0.146	<0.001 (+)
Cattle, above median number	0.167	0.053 (+)	0.003 (+)	0.418	0.305	<0.001 (+)
Cattle, number owned ^c^	0.003 (+)	0.001 (+)	<0.001 (+)	0.137	0.070 (+)	<0.001 (+)
*(b) Breastfed children*						
Month of dietary assessment, May	<0.001 (+)	<0.001 (+)	0.655	0.149	0.458	0.021
Child age	<0.001 (+)	<0.001 (+)	0.043 (+)	0.224	0.158	0.152
Sex of child, female	0.196	0.998	0.425	0.929	0.865	0.304
Child height-for-age Z-score	0.518	0.192	0.623	0.256	0.588	0.372
Maternal formal education, yes	0.673	0.517	0.460	0.134	0.326	0.680
Sex of household head, female	0.656	0.518	0.559	0.513	0.106	0.113
Number of household members	0.075 (+)	0.142	0.361	0.591	0.391	0.018 (+)
Language group, Sukuma	0.015 (+)	0.103	0.002 (+)	0.260	0.127	<0.001 (+)
Household domestic asset index ^c,d^	<0.001 (+)	0.002 (+)	<0.001 (+)	0.162	0.214	<0.001 (+)
Chickens owned, yes	0.012 (+)	0.004 (+)	0.008 (+)	0.104	0.242	0.013 (+)
Chickens, above median number	0.005 (+)	<0.001 (+)	0.004 (+)	0.162	0.178	0.003 (+)
Chickens, number owned ^c^	0.003 (+)	<0.001 (+)	<0.001 (+)	0.016 (+)	0.104	0.008 (+)
Cattle owned, yes	0.004 (+)	0.025 (+)	<0.001 (+)	0.181	N/A	<0.001 (+)
Cattle, above median number	<0.001 (+)	0.020 (+)	<0.001 (+)	0.289	0.704	<0.001 (+)
Cattle, number owned ^c^	<0.001 (+)	0.004 (+)	<0.001 (+)	0.182	0.048 (+)	<0.001 (+)
*(c) Non-breastfed children*						
Month of dietary assessment, May	<0.001 (+)	<0.001 (+)	0.003 (+)	0.849	0.834	<0.001 (+)
Child age	0.138	0.023 (+)	0.884	0.163	0.045 (−)	0.950
Sex of child, female	0.123	0.180	0.015 (−)	0.799	0.328	0.288
Child height-for-age Z-score	0.044 (+)	0.060 (+)	0.568	0.434	0.976	0.323
Maternal formal education, yes	0.165	0.237	0.332	0.536	0.968	0.212
Sex of household head, female	0.307	0.892	0.565	0.482	0.266	0.082 (−)
Number of household members	0.789	0.709	0.924	0.080 (+)	0.185	0.116
Language group, Sukuma	0.289	0.063 (+)	0.003 (+)	0.231	0.751	<0.001 (+)
Household domestic asset index ^c,d^	0.667	0.098 (+)	0.287	0.365	0.075 (+)	0.004 (+)
Chickens owned, yes	0.215	0.119	0.375	0.402	0.654	0.145
Chickens, above median number	0.725	0.097 (+)	0.169	0.998	0.432	0.021 (+)
Chickens, number owned ^c^	0.361	0.025 (+)	0.056 (+)	0.521	0.103	0.007 (+)
Cattle owned, yes	0.491	0.111	0.090 (+)	0.600	0.629	<0.001 (+)
Cattle, above median number	0.768	0.531	0.051 (+)	N/A	0.554	<0.001 (+)
Cattle, number owned ^c^	0.780	0.124	0.024 (+)	0.846	0.517	<0.001 (+)

^a^ Generalized linear mixed models using binomial distribution, allowing for geographic clustering and longitudinal data; ^b^ Binomial totals of 10 for women (MDD-W) and 7 for children (IYCMDD); ^c^ log-transformed variables used to minimize excessive influence of large numbers; ^d^ Cattle and chickens excluded from HDAI, evaluated as separate predictor variables.

**Table A3:** Univariable models evaluating the significance of predictor variables for height-for-age Z-scores (HAZ) ^a^, probability of stunting (HAZ < −2) ^b^ and diarrhea frequency ^b^ in children, showing *p*-values and the direction of associations (+/−). Grey shading indicates all suggestive associations (*p* < 0.1).

Predictor Variables	HAZ	Stunting	Diarrhea
Child age	<0.001 (−)	<0.001 (+)	<0.001 (−)
Sex of child, female	0.006 (+)	0.003 (−)	0.681
Diarrhea frequency	0.004 (−)	0.558	N/A
Height-for-age Z-score	N/A	N/A	0.641
Month of data collection, May ^c^	<0.001 (−)	0.011 (+)	0.031 (−)
Sex of household head, female	0.126	0.495	0.995
Number of household members	0.761	0.504	0.808
Maternal formal education, yes	0.794	0.305	0.351
Household language group, Sukuma	<0.001 (+)	0.001 (−)	0.512
Improved water source	0.312	0.627	0.298
Improved toilet facility	0.555	0.945	0.618
Household domestic asset index			
Livestock and non-livestock assets ^d^	0.205	0.061 (−)	0.072 (−)
Non-livestock assets only ^d^	0.076 (+)	<0.001 (−)	0.828
Non-livestock assets only, quintiles	<0.001 (+)	<0.001 (−)	0.558
Livestock			
Livestock owned, yes	0.086 (−)	0.368	0.327
“Livestock ladder” ^e^	0.134	0.467	0.259
Chickens owned, yes	0.214	0.925	0.128
Chickens, above median	0.002 (−)	0.109	0.479
Chickens, number owned ^d^	0.007 (−)	0.424	0.252
Chickens, location of overnight housing	0.651	0.692	0.101
Sheep or goats owned, yes	0.618	0.919	0.302
Sheep or goats, above median	0.121	0.035 (−)	0.398
Sheep or goats, number owned ^d^	0.100 (+)	0.260	0.513
Cattle owned, yes	0.392	0.541	0.046 (−)
Cattle, above median	0.340	0.060 (−)	0.385
Cattle, number owned ^d^	0.125	0.223	0.151
Children’s diet, previous 24 h			
ASF consumption, yes	0.075 (−)	0.405	0.367
Chicken meat consumption, yes	0.181	0.324	0.050 (−)
Other meat or fish consumption, yes	0.001 (−)	0.531	0.473
Egg consumption, yes	0.587	0.814	0.584
Milk consumption, yes	0.084 (+)	0.554	0.042 (+)

^a^ Linear mixed models, allowing for geographic clustering and longitudinal data; ^b^ Generalized linear mixed models using binomial distribution, allowing for geographic clustering and longitudinal data; ^c^ Two data collection months: May and November. Rainfall typically occurs between November and April in this area.; ^d^ log-transformed variables used to minimize excessive influence of large numbers; ^e^ Levels of livestock ownership (the “livestock ladder”): (1) none, (2) chickens only, (3) small ruminants +/− chickens, no cattle, (4) cattle +/− chickens and small ruminants.

## References

[1] Thornton P.K., Herrero M. (2015). Adapting to climate change in the mixed crop and livestock farming systems in sub-Saharan Africa. Nat. Clim. Chang..

[2] Altieri M.A., Nicholls C.I., Henao A., Lana M.A. (2015). Agroecology and the design of climate change-resilient farming systems. Agron. Sustain. Dev..

[3] Seo S.N. (2010). Is an integrated farm more resilient against climate change? A micro-econometric analysis of portfolio diversification in African agriculture. Food Policy.

[4] Murphy S.P., Allen L.H. (2003). Nutritional importance of animal source foods. J. Nutr..

[5] Neumann C.G., Harris D.M., Rogers L.M. (2002). Contribution of animal source foods in improving diet quality and function in children in the developing world. Nutr. Res..

[6] Randolph T.F., Ruel M., Schelling E., Grace D., Nicholson C.F., Leroy J.L., Cole D.C., Demment M.W., Omore A., Zinsstag J. (2007). Invited review: Role of livestock in human nutrition and health for poverty reduction in developing countries. J. Anim. Sci..

[7] Penakalapati G., Swarthout J., Delahoy M.J., McAliley L., Wodnik B., Levy K., Freeman M.C. (2017). Exposure to animal feces and human health: A systematic review and proposed research priorities. Environ. Sci. Technol..

[8] Zambrano L.D., Levy K., Menezes N.P., Freeman M.C. (2014). Human diarrhea infections associated with domestic animal husbandry: A systematic review and meta-analysis. Trans. R. Soc. Trop. Med. Hyg..

[9] Crane R.J., Jones K.D., Berkley J.A. (2015). Environmental enteric dysfunction: An overview. Food Nutr. Bull..

[10] Mbuya M.N., Humphrey J.H. (2016). Preventing environmental enteric dysfunction through improved water, sanitation and hygiene: An opportunity for stunting reduction in developing countries. Matern. Child Nutr..

[11] Ngure F.M., Reid B.M., Humphrey J.H., Mbuya M.N., Pelto G., Stoltzfus R.J. (2014). Water, sanitation, and hygiene (WASH), environmental enteropathy, nutrition, and early child development: Making the links. Ann. N. Y. Acad. Sci..

[12] Harper K.M., Mutasa M., Prendergast A.J., Humphrey J., Manges A.R. (2018). Environmental enteric dysfunction pathways and child stunting: A systematic review. PLoS Negl. Trop. Dis..

[13] Fierstein J.L., Eliasziw M., Rogers B.L., Forrester J.E. (2017). Nonnative cattle ownership, diet, and child height-for-age: Evidence from the 2011 Uganda Demographic and Health Survey. Am. J. Trop. Med. Hyg..

[14] Glatz P., Pym R. (2013). Poultry housing and management in developing countries. Poultry Development Review.

[15] Guèye E.F. (1998). Village egg and fowl meat production in Africa. World’s Poult. Sci. J..

[16] Aini I. (1990). Indigenous chicken production in South-East Asia. World’s Poult. Sci. J..

[17] Sonaiya E.B. (2004). Direct assessment of nutrient resources in free-range and scavenging systems. World’s Poult. Sci. J..

[18] Bagnol B. (2009). Gender issues in small-scale family poultry production: Experiences with Newcastle Disease and Highly Pathogenic Avian Influenza control. World’s Poult. Sci. J..

[19] Guèye E.F. (2005). Gender aspects in family poultry management systems in developing countries. World’s Poult. Sci. J..

[20] Guèye E.F. (2000). Women and family poultry production in rural Africa. Dev. Pract..

[21] Ruel M.T., Alderman H. (2013). Nutrition-sensitive interventions and programmes: How can they help to accelerate progress in improving maternal and child nutrition?. Lancet.

[22] World Bank (2007). From Agriculture to Nutrition: Pathways, Synergies and Outcomes.

[23] Headey D., Hirvonen K. (2016). Is exposure to poultry harmful to child nutrition? An observational analysis for rural Ethiopia. PLoS ONE.

[24] Headey D., Nguyen P., Kim S., Rawat R., Ruel M., Menon P. (2017). Is exposure to animal feces harmful to child nutrition and health outcomes? A multicountry observational analysis. Am. J. Trop. Med. Hyg..

[25] Hetherington J.B., Wiethoelter A.K., Negin J., Mor S.M. (2017). Livestock ownership, animal source foods and child nutritional outcomes in seven rural village clusters in Sub-Saharan Africa. Agric. Food Secur..

[26] Schmidt W.P., Boisson S., Routray P., Bell M., Cameron M., Torondel B., Clasen T. (2016). Exposure to cows is not associated with diarrhoea or impaired child growth in rural Odisha, India: A cohort study. Epidemiol. Infect..

[27] Headey D., Hirvonen K. (2015). Exploring Child Health Risks of Poultry Keeping in Ethiopia: Insights from the 2015 Feed the Future Survey. Essp ii Research Note 43.

[28] Mosites E.M., Rabinowitz P.M., Thumbi S.M., Montgomery J.M., Palmer G.H., May S., Rowhani-Rahbar A., Neuhouser M.L., Walson J.L. (2015). The relationship between livestock ownership and child stunting in three countries in Eastern Africa using national survey data. PLoS ONE.

[29] Chilonda P., Otte J. (2006). Indicators to monitor trends in livestock production at national, regional and international levels. Livest. Res. Rural Dev..

[30] Jahnke H.E. (1982). Livestock Production Systems and Livestock Development in Tropical Africa.

[31] Dolberg F. (2001). A livestock development approach that contributes to poverty alleviation and widespread improvement of nutrition among the poor. Livestock Res. Rural Dev..

[32] Maass B.L., Chiuri W.L., Zozo R., Katunga-Musale D., Metre T.K., Birachi E., Vanlauwe B., Asten P.V., Blomme G. (2013). Using the ‘livestock ladder’ as a means for poor crop–livestock farmers to exit poverty in Sud Kivu province, eastern DR Congo. Agro-Ecological Intensification of Agricultural Systems in the African Highlands.

[33] Todd H. (1998). Women climbing out of poverty through credit; or what do cows have to do with it?. Livest. Res. Rural Dev..

[34] Akinola L.A.F., Essien A. (2011). Relevance of rural poultry production in developing countries with special reference to Africa. World’s Poult. Sci. J..

[35] Alders R.G., Pym R.A.E. (2009). Village poultry: Still important to millions, eight thousand years after domestication. World’s Poult. Sci. J..

[36] Doran M.H., Low A.R.C., Kemp R.L. (1979). Cattle as a store of wealth in Swaziland: Implications for livestock development and overgrazing in Eastern and Southern Africa. Am. J. Agric. Econ..

[37] Moll H.A.J. (2005). Costs and benefits of livestock systems and the role of market and nonmarket relationships. Agric. Econ..

[38] Alders R., Aongola A., Bagnol B., de Bruyn J., Kimboka S., Kock R., Li M., Maulaga W., McConchie R., Mor S. (2014). Using a one health approach to promote food and nutrition security in Tanzania and Zambia. GRF Davos Planet@Risk.

[39] Lema M., Majule A. (2009). Impacts of climate change, variability and adaptation strategies on agriculture in semi arid areas of Tanzania: The case of Manyoni District in Singida Region, Tanzania. Afr. J. Environ. Sci. Technol..

[40] FAO and FHI 360 (2016). Minimum Dietary Diversity for Women: A Guide for Measurement.

[41] WHO (2013). Diarrhoeal Disease (Fact Sheet No. 330).

[42] WHO Multicentre Growth Reference Study Group (2006). WHO Child Growth Standards based on length/height, weight and age. Acta Pædiatr. Suppl..

[43] WHO (2009). WHO AnthroPlus for Personal Computers Manual: Software for Assessing Growth of the World’s Children and Adolescents.

[44] WHO (2008). Indicators for Assessing Infant and Young Child Feeding Practices. Part 1: Definitions.

[45] Bandoh D.A., Kenu E. (2017). Dietary diversity and nutritional adequacy of under-fives in a fishing community in the central region of Ghana. BMC Nutr..

[46] Alkire S., Meinzen-Dick R.S., Peterman A., Quisumbing A.R., Seymour G., Vaz A. (2013). The Women’s Empowerment in Agriculture Index. Oxford Poverty and Human Development Initiative Working Paper No. 58.

[47] Njuki J., Poole J., Johnson N., Baltenweck I., Pali P., Lokman Z., Mburu S. (2011). Gender, Livestock and Livelihood Indicators.

[48] Mabilia M. (1996). Beliefs and practices in infant feeding among the Wagogo of Chigongwe (Dodoma Rural District), Tanzania: I. Breastfeeding. Ecol. Food Nutr..

[49] Selemani I.S., Eik L.O., Holand Ø., Ådnøy T., Mtengeti E., Mushi D. (2012). The role of indigenous knowledge and perceptions of pastoral communities on traditional grazing management in north-western Tanzania. Afr. J. Agric. Res..

[50] WHO, UNICEF (2006). Core Questions on Drinking-Water and Sanitation for Household Surveys.

[51] Allegretti A. (2017). ‘Being Maasai’ in markets and trade: The role of ethnicity-based institutions in the livestock market of northern Tanzania. Nomadic Peoples.

[52] Savy M., Martin-Prevel Y., Traissac P., Eymard-Duvernay S., Delpeuch F. (2006). Dietary diversity scores and nutritional status of women change during the seasonal food shortage in rural Burkina Faso. J. Nutr..

[53] Buza J.J., Mwamuhehe H.A., Alders R.G., Spradbrow P.B. (2001). Country report: Tanzania. Proceedings of SADC Planning Workshop on Newcastle Disease Control in Village Chickens, Maputo, Mozambique, 6–9 March 2000.

[54] Ministry of Health Community Development Gender Elderly and Children [Tanzania Mainland] (MoHCDGEC), Ministry of Health [Zanzibar] (MoH) (2016). Tanzania Demographic and Health Survey and Malaria Indicator Survey 2015–16.

[55] Guèye E.F. (2002). Employment and income generation through family poultry in low-income food-deficit countries. World’s Poult. Sci. J..

[56] Pym R.A.E., Guerne Bleich E., Hoffman I. (2006). The Relative Contribution of Indigenous Chicken Breeds to Poultry Meat and Egg Production and Consumption in the Developing Countries of Africa and Asia. Presentation at XII European Poultry Conference, Verona, Italy, 10–14 September 2006.

[57] de Bruyn J., Bagnol B., Darnton-Hill I., Maulaga W., Thomson P.C., Alders R. (2017). Characterising infant and young child feeding practices and the consumption of poultry products in rural Tanzania: A mixed methods approach. Matern. Child Nutr..

[58] Udo H.M.J., Aklilu H.A., Phong L.T., Bosma R.H., Budisatria I.G.S., Patil B.R., Samdup D., Bebe B.O. (2011). Impact of intensification of different types of livestock production in smallholder crop-livestock systems. Livest. Sci..

[59] Neumann C.G., Murphy S.P., Gewa C., Grillenberger M., Bwibo N.O. (2007). Meat supplementation improves growth, cognitive, and behavioral outcomes in Kenyan children. J. Nutr..

[60] Darwish W.S., Ikenaka Y., Nakayama S.M.M., Ishizuka M. (2014). An overview on mycotoxin contamination of foods in Africa. J. Vet. Med. Sci..

[61] Gizachew D., Szonyi B., Tegegne A., Hanson J., Grace D. (2016). Aflatoxin contamination of milk and dairy feeds in the Greater Addis Ababa milk shed, Ethiopia. Food Control.

[62] Knight-Jones T., Hang’ombe M., Songe M., Sinkala Y., Grace D. (2016). Microbial contamination and hygiene of fresh cow’s milk produced by smallholders in Western Zambia. Int. J. Environ. Res. Public Health.

[63] Budlender D. (2003). The debate about household headship. Soc. Dyn..

[64] Rosenhouse Persson S. (1989). Identifying the Poor: Is “Headship” a Useful Concept? Living Standards Measurement Study Working Paper No. 58.

[65] Kennedy E., Haddad L. (1994). Are pre-schoolers from female-headed households less malnourished? A comparative analysis of results from Ghana and Kenya. J. Dev. Stud..

[66] Cronk L. (1991). Preferential parental investment in daughters over sons. Hum. Nat..

[67] Miller B.D. (1997). Social class, gender and intrahousehold food allocations to children in South Asia. Soc. Sci. Med..

[68] Espo M., Kulmala T., Maleta K., Cullinan T., Salin M.L., Ashorn P. (2002). Determinants of linear growth and predictors of severe stunting during infancy in rural Malawi. Acta Paediatr..

[69] Ngare D.K., Muttunga J.N. (1999). Prevalence of malnutrition in Kenya. East Afr. Med. J..

[70] Wamani H., Åstrøm N., Peterson S., Tumwine J., Tylleskär T. (2007). Boys are more stunted than girls in sub-Saharan Africa: A meta-analysis of 16 Demographic and Healthy Surveys. BMC Pediatr..

[71] Garenne M. (2003). Sex differences in health indicators among children in African DHS surveys. J. Biosoc. Sci..

[72] Dumville J.C., Torgerson D.J., Hewitt C.E. (2006). Reporting attrition in randomised controlled trials. Br. Med. J..

[73] Zafar S.N., Luby S.P., Mendoza C. (2009). Recall errors in a weekly survey of diarrhoea in Guatemala: Determining the optimal length of recall. Epidemiol. Infect..

[74] Tomlinson M., Swartz L., Landman M. (2006). Insiders and outsiders: Levels of collaboration in research partnerships across resource divides. Infant Ment. Health J..

[75] Webb P., Kennedy E. (2014). Impacts of agriculture on nutrition: Nature of the evidence and research gaps. Food Nutr. Bull..

